# Eclectic Article on Pulmonary Tubercles or Tubercular Phthisis

**Published:** 1830-01-01

**Authors:** 


### ON PULMONARY TUBERCLES, OR TUBERCULAR PHTHISIS.

[Eclectic.]

1. CLINICAL OBSERVATIONS ON PHTHISIS. By *William Stoker*, M. D. [Dublin Transactions.]
2. OF TUBERCLES IN THE LUNGS, OR PHTHISIS PULMONALIS. By *M. Laennec*. (2d edition.)
3. ON PULMONARY PHTHISIS. By *M. Andral*, Junr. [Dictionnaire de Medicine, Vol. XVI.
4. A PRELIMINARY DISSERTATION ILLUSTRATIVE OF A NEW SYSTEM OF PULMONARY PATHOLOGY, &c. By *P. Myddleton*, M. D.
5. REPORT OF MEDICAL CASES, &c. By *Dr. Bright*. 4to. with plates, 1827.

At the head of this article we have indicated four or five of the latest monographs on that destructive disease, TUBERCULAR PHTHISIS—two of them by two of the most distinguished pathologists which France has produced—and one of them by our countryman Dr. Stoker, who as physician to the Meath Hospital, has had ample opportunities of corroborating or correcting the observations of his illustrious predecessors in this line of research. We have thought that it might not be a useless labour to lay before the profession a succinct account of the present state of our knowledge, etiological, pathological, and therapeutical, respecting one of the greatest scourges of the human race—and, for this purpose, we shall principally avail ourselves of M. Andral's dissertation, which forms one of the ablest articles in the dictionary of medicine, now nearly completed by a galaxy of eminent men on the continent. We conceive that one great use of a medical journal is, from time to time, to bring within the reach of all classes of medical readers, a comprehensive yet concentrated view of the principal diseases which form the objects of our solicitude in actual practice; and thus to keep up as great an equilibrium of information as possible, among those who have, and those who have not, the means of extended investigation, or access to the various works that flow from the medical press. We believe indeed that, multiplied as medical journals have become in this and other countries, there is yet a field for one that should be entirely dedicated to the abovementioned purpose—a series of SYNTHETICAL or ECLECTIC ARTICLES on the practice of medicine generally. Till such time as a Journal of this kind is attempted, we shall continue to dedicate a portion of our own to this specific pursuit; believing, from the encouragement we have hitherto had, that such a line of labour will not be unacceptable to our readers at large.

M. Andral coincides with Laennec and Louis, in restricting the term pulmonary consumption to *tubercular* phthisis. The same view is taken by Dr. Stoker, who regrets to say that "the term phthisis is one too often used (at least in this country) without an accurate idea of its nature." Thus Bayle includes under the definition of consumption, six species of pulmonary phthisis—tubercular, granular, melanose, ulcerous, calculous, and cancerous. That life is occasionally destroyed by one or other of the last five species, we do not deny; but the tubercular species is the one which meets us one hundred times more frequently than all the others put together. It is therefore quite enough for our purpose in this article—and we agree with the talented writers above-mentioned, that it would prevent a great deal of confusion if, by the term pulmonary consumption, we always meant tubercular phthisis.

### I. STATE OF THE RESPIRATORY APPARATUS IN THE DIFFERENT PHASES OF PULMONARY PHTHISIS.

The lesions of structure are of two kinds—one permanent, namely, the tubercles themselves—the other, variable, consisting of the divers alterations that take place in the surrounding lung, and which give rise to many of the most prominent symptoms of the disease. The tubercles though constant in their existence, undergo changes in their condition, at different periods of the malady—changes which are announced during life by corresponding symptoms. They are hard or crude in the first period—soft in the second—and in a course of evacuation in the third. Laennec regards the "granulations of the lungs," described by Bayle, as the rudimentary condition of pulmonary tubercles—and M. Louis adopts the same opinion. M. Andral, in his "*Clinique Medicale*," has endeavoured to prove that these pulmonary granulations are an accidental tissue, *sui generis*, the product of pneumonia, and independent of tuberculation. He gives very cogent reasons for this conclusion, and it will probably be found to be the correct one. Crude tubercles present themselves under two forms, not essentially different in any thing else than *form*. The tubercular matter is sometimes infiltrated *among* the pulmonary tissue—sometimes deposited in globular masses by itself. The infiltrated tuberculation is very often confounded with that state of lung which results from chronic inflammation.

After a longer or shorter period, a process of mollescence commences in the centre, and gradually extends to the periphery of the tubercle. Then the circumjacent parenchyma of the lung inflames—a bronchial tube becomes perforated—the tubercular matter issues through this aperture and is evacuated by coughing—and thus a cavity or cavern is formed. If the tubercle be situated in the immediate vicinity of the pleura pulmonalis, the ulcerative inflammation may open a communication with the cavity of the chest, instead of the bronchia, and the tubercular matter is evacuated into that cavity. The cavern formed by the evacuation of tubercular matter, in either way, is often much larger than the original tubercle. Sometimes a whole lobe of lung is thus transformed into one vast cavern, the parietes of which are only a few lines in thickness. This, however, proceeds from the re-union of several neighbouring cavities, and the consequent destruction of the intervening parenchymatous structure. There is then, as was justly supposed by the ancients, a veritable *ulceration* of the lungs. The internal surface of these caverns or excavations, is generally of a vivid red colour, excepting under certain circumstances where there is a tendency to cicatrization. It is almost always lined by a whitish matter resembling a false membrane, often divisible into layers, and formed, no doubt, by the coagulable lymph thrown out from the vessels. On this internal surface are seen blood-vessels of considerable size, as also bronchia, which appear abruptly divided where they enter the excavation. Across these caverns are seen stretching bands of condensed cellular substance dividing them into different compartments. In these bands, vessels of considerable size are sometimes found running, and from these, when ruptured, effusions of blood take place. These bands, as well as the internal surface of the parietes of the excavation, are sometimes found in a state of gangrene, and exhaling the most intolerable odour. The usual contents of these excavations, however, are a puriform fluid resembling that which issues from scrofulous abscesses, and is evidently secreted from the parietes themselves. Even the little rounded masses of what is called tuberculous matter, are unequivocally secreted, long after the original tubercle is completely evacuated—a fact of some consequence in the etiology of tubercles. Blood is sometimes found in these caverns, as also calculous concretions. Fragments of parenchymatous structure are also occasionally seen swimming in the fluid contents of the cavern, and are expectorated by coughing. Sometimes these caverns are found completely empty.

These excavations once formed, three different conditions may result. The cavities may continue to enlarge—to remain stationary—or to cicatrize. In order that this last event may obtain, it is essential that the internal surface of the excavation should secrete a matter different from pus—in short, a matter capable of being transformed into a fibrous, cartilaginous, or serous tissue, the same as forms in apoplectic cysts. The progressive changes which take place in the different phases of this salutary process have been carefully ascertained by the unwearied observations and dissections of Laennec, Andral, Louis, and many other continental pathologists of the greatest celebrity.

The part most commonly affected with tubercles is the superior lobe of each lung. In general, tubercles are found in both lungs at the same time, but often more plentiful in one side than in the other. Instances, though very rare, are on record where one lung was perfectly sound, and the other studded with excavations.

The parenchyma situated between the tuberculous masses or caverns, presents different appearances. Sometimes it is healthy, especially where the tubercles are crude—rarely so around excavations. Sometimes the parenchyma is found in a state of acute or chronic inflammation, with yellow, grey, or black induration. This inflammation often obtains but to a very limited extent around the tubercles, and therefore may not be recognized during life. It is rendered highly probable that tubercles are capable of a transformation into calculous masses by a species of desiccation.

The following modification of tuberculation (somewhat different from Andral,) is given by Dr. Bright, under the head of "PHTHISIS PULMONALIS," in his recent work.

"It will be seen in the following Cases, that the tubercular deposit assumes two totally different forms; so that were it not for the fact of their occurring so frequently together in the same individual, in the same lung, and even in the same lobe of that lung, we should scarcely be authorized in considering them the result of the same, or even of analogous morbid actions. It will be seen that sometimes a portion of the lung, varying from the size of a nutmeg to the greater part of a whole lobe, has become of a dense semicartilaginous consistence, has assumed a permanent blueish gray colour, and is nearly translucent; that this has gone on gradually to become yellow in spots, to soften, and to form abscesses of a sluggish character, finding their way in time to some unobstructed bronchus, and discharging themselves by expectoration. The cavity becomes larger and larger by the suppuration of the internal surface, which appears like a secretion from a vascular tissue, with which it is surrounded; this however is not exactly the case, as there is reason to think that *the cavity actually enlarges*, and therefore that the internal surface must waste, and be renewed by a fresh membrane forming beneath. In other parts of the same lung a very different process has often been going on; and a number of minute bodies, not larger than the smallest shot, have been deposited more or less thickly throughout the substance. The lung may scarcely be altered in its appearance; but on pressing it, hard unyielding bodies are distinctly felt; these in a short time enlarge, and when still not larger than small peas, begin to suppurate at the centre; or more frequently these small miliary tubercles join in clusters, either forming masses of themselves, or inclosing a piece of the lung, which becomes hard and blue and semi-transparent, and either runs into suppuration together with the small tubercles; or, if no suppuration takes place towards the centre, becomes completely separated, and forms a slough in the midst of the cavity, which is surrounded by the tubercular matter in the form of a cyst. These are the two extreme points of the processes which take place in genuine tubercular phthisis; and they admit of many modifications, more particularly when combined with the effusion of fibrin which attends simple inflammation." 149.

The larynx, the trachea, and the bronchia are generally changed, more or less, in structure, during the progress of tuberculation—though sometimes the whole of the air-passages are found in a state of perfect integrity. The changes observable are the products of inflammation—redness, thickening, ulceration of the mucous membrane, with occasional development of tubercles in the subjacent cellular tissue, especially in the larynx. These have been supposed to be capable of producing all the phenomena of pulmonary consumption; but such a case, M. Andral thinks, is very rare, independent of tubercles in the lungs themselves.

II. Causes of Phthisis. The etiology of tubercles in the lungs is a subject as interesting as it is, unfortunately, obscure. "Inflammation," says M. Andral, "appears to me to play a more important part in the production of tubercles than has been assigned to it by the school of Bayle; but, on the other hand, this inflammation will not satisfactorily account for the formation of the said bodies, notwithstanding the doctrine of M. Broussais, and his disciples." M. Andral thinks himself justified in concluding, contrary to the opinions of Bayle, Laennec, Louis, and we may add Baron, that, in the great majority of cases where tubercles have invaded the pulmonary tissue, there has been previous congestion or inflammation of the said tissue. He draws this conclusion from various practical observations:—thus, phthisis very often succeeds pneumonia, there having been no previous symptoms of tubercles in the lungs. It is true that the opposite party will argue that, in these cases, there were latent tubercles in the lungs, and that they were only called into an active state by the inflammation. In the second place, it may be observed that the ancients and many of the moderns generally admitted that phthisis is a frequent sequence of hæmoptysis. The school of Bayle has reversed this precept, and asserted that every case of hæmoptysis, which takes place in an individual who afterwards presents symptoms of tubercles, is the product of these tubercles but never the cause of them. M. Andral, while he admits that hæmorrhage from the lungs is often merely symptomatic of tubercles there, labours to prove that, in some cases at least, the sanguineous congestion is the cause of the tubercular formation. We think his facts, on this point, are unsatisfactory, and his arguments rather weak.

The fact of tubercular phthisis being often preceded by neglected catarrhal inflammation, is equivocal, for the same reasons as have been urged above. M. Andral still thinks that these protracted inflammations of the bronchial mucous membrane tend to develop, if not create, tubercles—and in this opinion we apprehend he will be joined by most observant physicians. The same illustrious pathologist attributes great importance, in the production of tubercles, to partial or local inflammations in the lungs. In these portions he has seen immense crops of tubercles, while in the uninflamed portions there were none. He cannot believe that the tubercles were the cause of the partial inflammation, as Bayle and others have urged.

"In fine," says he, "the observation of symptoms, the dissection of bodies, reasoning founded on analogy, appear to demonstrate that, in the great majority of cases, the development of pulmonary tubercles is preceded by sanguineous congestions, in various degrees, while the opposite circumstances form merely the exceptions."

If we examine into the causes which are generally supposed to favour the development of phthisis, we shall find that they all have a tendency to produce congestion in the lungs—for example, narrowness of the chest, which often leads to hæmoptysis, the frequent precursor of phthisis. Atmospheric vicissitudes—excesses of every kind—even the depressing and other strong passions, have all a tendency to throw an inordinate proportion of blood on the internal organs, and produce pulmonary congestion. The suppression of accustomed discharges, as the menses—the drying up of old ulcers—the retropulsion of cutaneous affections, are all known to be not uncommon precursors of tubercular consumption, and it is unnecessary to observe that they are very likely to produce previous congestion in the lungs. Again, we all know how often tubercular phthisis becomes developed after acute diseases, as fevers, measles, small-pox, and the various phlegmasiæ. These, of course, cannot take place without effecting more or less of pulmonary congestion. From all these considerations, M. Andral holds it as proved that, as, at the commencement of all morbid secretions, so in the case of tubercular secretion, there is a state of sanguineous congestion in the vessels of the tissue where the tubercles are deposited. "But this congestion will not, of itself, produce tubercles:—there must be a specific disposition to such disease." This would go pretty nearly to the conclusion that the rudiments or ova of tubercles are born with us; but that congestion or inflammation of the parts containing these ova, exerts a strong influence in their development. In respect to the intimate nature of this specific tubercular predisposition, we know very little. Observation teaches us that, in scrofulous constitutions, whether hereditary or acquired, tubercles are much more readily developed than in other constitutions. Unfortunately, however, there is no constitution exempt from visitations of this dreadful disease! Tubercles have been found in the fœtus in utero—in every period of childhood—in every age till that of extreme senectitude. Laennec saw a man perish by tubercular phthisis at the age of 99 years. "Too many proofs exist of the hereditary character of this disease." M. Andral does not believe that the hereditary taint can depend on germs of tubercles transmitted from parent to progeny—but on a *simple predisposition.* We think the former equally probable and conceivable as the latter supposition. Neither of them is susceptible of direct proof.

III. SYMPTOMATOLOGY. Hacknied as this part of the subject may appear to the sciolist, it is deserving of extreme attention and study by the zealous and conscientious practitioner. Every tyro thinks he can recognize tubercles in the lungs, or tubercular consumption, at a glance ; whereas it is very often extremely difficult to distinguish the disease in that stage in which any great hope remains of cure, notwithstanding the aids which semeiology has received from pathology, auscultation, percussion, &c.

Phthisis is divided by systematic writers into three periods, stages, or degrees, to each of which a group of characteristic symptoms is attached. This, like many other arrangements, has very little foundation in nature or fact. Thus, in many cases, we find the symptoms of one stage or degree united with those of another stage. In some people we find emaciation, fever, rigors, puriform expectoration ; and yet auscultation can discover no excavation. In others, the existence of this excavation is unequivocally proved by auscultation, while there is no appearance of emaciation, &c. M. Andral thinks it much more scientific and useful to carefully analyse each symptom of the disease, and trace it to its source.

COUGH is one of the most constant attendants on tubercles in the lungs. It is owing to irritation of the bronchia, and increases or diminishes with that irritation. In the early periods of the disease, it is often only present at intervals :—in some cases, after having existed, with more or less intensity, for a time, the cough ceases entirely, and the patient wastes away and dies without even a catarrhal symptom. "In such instances," says M. Andral, "I have found crude or softened tubercles thickly disseminated through the substance of the lungs, as well as through the mucous membrane of the bronchia." M. Louis has met with similar cases. The cough, in incipient phthisis, has been characterised as short and dry. This kind of cough certainly is very common at such period—and it may remain so till the patient's death —a circumstance owing either to non-mollescence of the tubercles, or to paucity of secretion from the bronchial mucous membrane. But, in many cases, the cough, from the very beginning, is moist, and comes in paroxysms. In numerous instances, among children, our author has seen it take the form of pertussis. Not unfrequently the cough becomes less troublesome in proportion as the excavations are formed—a circumstance which is fortunate for the patient, but should not lull the practitioner into a delusive hope. This event seems to depend on a diminution of viscidity in the expectorated matter. From what has been said, it will be evident that there is nothing so specific in the character of the cough, as to throw much light on the diagnosis of tubercular phthisis.

The study of the EXPECTORATION has excited great interest in all ages. The existence of pus, or of the debris of tubercles, has been anxiously sought. Such researches sometimes lead to probabilities, and are so far useful—but they rarely end in any thing like certitude. In the early stage of phthisis, and before the tubercles have softened down, the expectoration is, of course, purely a secretion from the mucous membrane of the bronchia, and presents all the varieties of sputum seen in catarrhal inflammation, acute and chronic. Afterwards, when the tubercles begin to break down, we find, mixed with the bronchial mucus, a matter which appears to belong to softened tubercles, and which presents itself sometimes in the form of little globular clots, white and untenacious—sometimes in the form of striæ floating in the mucus. But these suspicious clots (grumeaux) may be nothing but a secretion from the tonsils—and the striæ may proceed entirely from the minute ramifications of the bronchia. Thus, so long as no excavations are yet formed in the lungs, the signs furnished by the expectoration are equivocal or even null. Are they unequivocal when the cavities *are* formed? In these cases, the tuberculous matter and the pus formed from the sides of the cavity, must exist in the expectoration, mixed with the mucus secreted from the bronchial mucous membrane, and we have only to distinguish these different products by their proper physical characters. But, alas! these characters are rendered extremely variable, *first* by the manner in which the bronchia communicate with the excavation—*secondly*, by the number, length, width of the bronchial tubes along which the expectoration travels before it reaches the trachea —*thirdly*, by the quantity and quality of the bronchial mucus with which the tuberculous matter is mixed—*fourthly*, by its longer or shorter sojourn in the bronchia. All the various kinds, colours, shapes and appearances of expectoration from tuberculated lungs and tuberculous cavities, have been so often seen in cases of simple chronic bronchitis, that they can no longer be regarded as affording any *positive* proof of the existence of tubercular phthisis. All that can be said is this:—among the various appearances in the expectoration, there are some that are much more frequently seen in cases of tubercular excavations than under any other circumstances. Such are the globular or rounded masses which are seen swimming in a liquid resembling a thick solution of gum in water. On the other hand, there are cases where tuberculous cavities exist, and yet where the expectoration is scanty, and apparently consisting of the common mucosities that attend the simplest bronchitis. Here we need not speak of chemical tests for tuberculous expectoration. They have long since been discharged as useless.

It sometimes happens that the contents of a large tubercle are evacuated all at once through a bronchial tube; in which case there is a great discharge of pus in which are seen swimming many of the globular clots already described. This is what Laennec has described under the term VOMICA.

The expectoration of phthisical subjects is, for the most part, inodorous—but it is sometimes very fetid, even in early stages of the disorder. This fetor, M. Andral thinks, may sometimes depend on gangrenous patches thrown off from the internal surface of the excavation—but it is also found to exist where there is no gangrene whatever. He has observed it in cases where there was no other disease than simple bronchitis.

The mucous or purulent expectoration in phthisis is often changed into a sanguineous discharge more or less considerable. Hæmoptysis and tubercles indeed are so often seen in connexion, that when we observe the one, we generally suspect the other. There are, however, many individuals who bring up considerable quantities of blood, at various intervals, from the lungs, and who never become phthisical. It is well known, on the other hand, that numbers die every year of phthisis, who never had hæmoptysis.

The discharge of blood from the lungs sometimes precedes the phthisical symptoms—sometimes takes place in the course of the disease—even a few days before death. The blood, in these cases, comes from three sources—the parenchyma of the lung—the bronchial mucous membrane—or the parietes of the excavation.

The RESPIRATION is but little incommoded in very many cases of phthisis, whether the tubercles be crude, softened, excavated, or even surrounded with a good deal of hepatised lung impermeable by the air. But if the phthisis be suddenly developed in the acute form, then the dyspnœa is considerable, and sometimes becomes the prominent symptom of the complaint, causing apprehension in the mind of the medical practitioner that there is organic disease of the heart. Bating this sudden development, we generally find the respiration more or less incommoded by all inflammatory attacks, by the process of digestion, the return of the menses, moral emotions. There are many, however, who have an habitual shortness of breath for many years before the unequivocal development of phthisis. Whether this be owing to nascent tubercles, or an habitual state of plethora in the vessels of the lungs, it is difficult to say.

PAIN is by no means a necessary accompaniment to the formation of tubercles. Those fixed or flying pains which phthisical patients sometimes complain of in different parts of the chest, appear referrible to sympathetic irritations of the pleura. A patient of M. Louis who complained of most violent pain in a certain point of the pleura during life, presented not the slightest trace of inflammation in that membrane on dissection.

FEVER is not found at all periods of tubercular phthisis. Early in the disease there is none; but in proportion as the lung becomes disorganized, we observe the accessions of fever;—at first, rare and erratic, but afterwards the paroxysms approaching each other, till at length there is fever every night. These accessions are characterized by an intense heat, which is not generally, nor even frequently preceded by rigor, or succeeded by perspiration, till the disease is more advanced. In the last stage, the fever is constant, with nocturnal exacerbations terminating towards morning in profuse perspiration, partial or general. Some phthisical patients, however, die, without having experienced any perspirations; and what is curious, there are cases where pulmonary tubercles produce neither cough, expectoration, nor dyspnœa, but merely a simple slow fever, which wastes the patient to a skeleton, and the cause of which often remains undiscovered till the last.

Such are the symptoms of tubercles in the lungs, and their fatal effects on the constitution. It must be evident that there are no pathognomonic symptoms among those described, on which we can depend, for ascertaining the precise nature of the malady—and therefore it is natural that we should have recourse to such other physical investigations as may enable us to strengthen our diagnosis and direct our prognosis. It is in this line of research that the moderns have greatly excelled their forefathers. They could not surpass them in faithful and accurate observation of external symptoms or phenomena; but they have, as it were, added another sense to those previously possessed.

PERCUSSION of the chest, in phthisical cases, furnishes the following information:—1mo. The sonoreity of the thoracic parietes may remain natural, when the pulmonary parenchyma surrounding the tubercles is sound—or at least permeable by the air drawn in :—and this will be the case whether the tubercles be crude, softened down, or even excavated. 2ndo. The natural sonoreity of the chest may be augmented, when a large excavation exists in the lungs near the surface—or when pulmonary emphysema, or pneumo-thorax obtains. The emaciation of phthisical patients contributes also to the natural sonoreity of the chest. 3tio. There may be a diminution of resonance in the chest, or a dull sound in one or more points. This indicates that the tubercles are agglomerated in considerable quantities —or that the parenchyma of the lungs around the tubercles is hepatized, (a circumstance more commonly the cause of the dull sound than the agglomeration of tubercle)—or, finally, that an effusion of fluid exists in the cavity of the pleura. When the dull sound, suppose under one of the clavicles, is produced by the presence of a large mass of tubercles, it may happen that, after the evacuation of the tuberculous matter, the sound returns. Often around a circumscribed point where the sound is very clear, on percussion, we find the sound quite dull. This indicates the existence of an excavation, surrounded by a portion of hepatised lung.

Auscultation, which, after all, is the foundation or essence of percussion, presents for study the following points:—1mo. Various modifications of the respiratory noise—2do. Several rattles or wheezes which supersede the respiratory murmur—3tio. That peculiar resonance of the voice called by Laennec pectoriloquy. The respiratory murmur remains natural when there is no other affection of lung than the presence of tubercles as yet crude. Under such circumstances, the respiration may even be more loud than natural, as if the presence of tubercles caused a kind of supplemental respiration in the contiguous sound lung. The breathing, however, may be rendered less distinct, or even null, if the tubercles be very much multiplied. From all these considerations it is manifest that auscultation furnishes no decisive means of ascertaining the presence of tubercles. If a great portion of pulmonary tissue becomes indurated, the inspired air only passes through the larger bronchial tubes, and then makes a loud noise, which is termed *bronchial* respiration. It is called *cavernous* respiration when the air passes freely from the larger bronchia into an excavation. The noise then resembles the blowing into an empty bottle, and has been called by Laennec the *amphoric* respiration.

The natural respiratory murmur is obscured, in many cases of tubercular phthisis, by different wheezing noises or rattles, which sometimes mask it entirely. These rattles exist in the bronchia or in the excavations—their numerous varieties depending chiefly on the quantity or quality of the matters contained in those parts—on the diameters of the cavities, their mode of communication with the bronchia, and the condition of the cavernous parietes. For minute information on these points, (which we do not, however, consider as of very great importance,) we must refer to the great work of Laennec.

When the Pectoriloquy is *unequivocally* heard, there can be no doubt of the existence of an excavation ; but it must be confessed that *perfect* pectoriloquism is rarely heard, even when the excavation exists, in consequence of the cavity being seldom evacuated, and several other circumstances. The absence therefore of pectoriloquism does not completely prove the absence of excavations. In general, however, the concomitant symptoms, joined to the auscultic phenomena, leave little doubt as to the state of parts in such cases.

There are many moral as well as physical phenomena presented by the phthisical patient, which guide the experienced practitioner in forming his diagnosis and prognosis. One of the most prominent and curious, in the former class, is the constant and universal endeavour of the phthisical patient to hide; or if he cannot hide, to disguise the real symptoms of his malady. The lawyer has not more difficulty in sifting out the truth from the lips of a culprit, than has the physician in getting at a confession of the symptoms of phthisis, as far as they are to be gathered from the statements of the patient. He will often seem to anticipate your thoughts and try to baffle your conclusions! Have you any pain in either of your sides? Yes; but it is only slight rheumatism which shifts about, and is of no consequence. Can you lie better on one side than on the other? Yes? but it is, I am sure, from *habit*, that I cannot lie on my left side. Are you thinner than you were a few months ago? Yes; but it is entirely owing to the low living which my doctor prescribed. Have you any perspirations at night? No—yes; but they are owing to the heat of the room, when I have them. Please to draw in a full breath. The phthisical patient will make such a tremendous gasp, that you would suppose he was going to draw in half a bushel of air—and yet a pint of that fluid will set him coughing violently, to his great mortification, as it is his constant endeavour to suppress his cough all the time you are examining him. If there be any glairy tough expectoration holding much air, the patient will be sure to have a bason of water ready for you to try whether the matter sinks or swims, he well knowing that the latter is to take place. But if the expectoration is dense heavy pus, it will be a great chance if any water can be got in the house. In short, no General ever more carefully guarded his positions from the reconnoissances of the enemy, than will the real phthisical patient conceal all his weak points from his physician. It is extremely difficult to account for this strong propensity to deceive, on the part of the phthisical patient; but so it is, and the young practitioner is frequently thrown off his guard in such examinations. These patients are particularly apt to attribute their cough to *stomach disorder;* and the bias of medical opinion having lately taken that turn, the deception has been greatly increased.

IV. Complications of Phthisis. It is seldom that an anatomical investigation of those who die of phthisis does not disclose other lesions of structure than pulmonary tubercles. Some of these are rare, and purely accidental—others very common, as inflammation of the parenchyma of the lungs, adhesions of the pleura, various degrees of inflammation in the larynx, digestive tube, &c. These last have been observed by M. Louis in four-fifths of the bodies he has examined. This distinguished physician, indeed, considers phthisis as a predisposing cause of gastric and intestinal inflammation. It is by no means uncommon to find a large growth of tubercles in the digestive tube of phthisical patients. In some cases, the disease appears to commence in the intestines, the lungs becoming tuberculated afterwards. Another organic lesion not unfrequently met with in phthisis, is very unaccountable—a greasy or fat state of the liver. M. Louis found this to be the case in two-thirds of the phthisical bodies that he opened. The swellings of lymphatic glands have been commonly recognized as connected with phthisis. A general disposition to the development of tubercles in various organs of the body, as well as in the lungs, is by no means uncommon. It is not a little curious that this almost universal tuberculation sometimes shews no symptom of such a state till death takes place, when the tubercles are unexpectedly found.

V. Progress—Duration—Termination. The progress of phthisis is generally continued—but sometimes it is observed to be intermittent. Thus individuals have been seen presenting, for a time, all the symptoms of phthisis, and afterwards getting apparently well, again to relapse, and again to recover. The ordinary or medium duration of phthisis is from six months to two years. In some cases it is protracted to a much greater length of time. Bayle asserts that the disease may last 40 years, in which case it would certainly be entitled to the term "slow consumption!" Our author himself has recorded the case of a man 76 years of age, who died of phthisis, having exhibited symptoms of that disease for thirty years previously. There are, on the other hand, numerous instances where phthisis has been so acute as to run its course and terminate fatally in less than a month. Dr. Stoker, in the work at the head of this article, observes that phthisis very often appeared in the course of a few days after the termination of fever, in those who were predisposed to the disease; and that in many of such cases, "the development and suppuration of tubercles were exceedingly rapid." "I have seen cases," says he, "in which scarcely any pulmonary symptoms existed before the occurrence of the fever, yet where death, from suppuration of tubercles in the lungs, took place so soon as eight days from the crisis." On dissection, the lungs were found every where penetrated by anfractuous cavities of recent formation. In some cases, death occurred before any suppuration of tubercles obtained—"the symptoms being those of the highest inflammatory fever;"—these instances, he observes were rare, but as the affection may be considered as *cute* phthisis, he has related some cases of the complaint. Sometimes, says M. Andral, the phthisis presents the usual symptoms in these rapid courses—the rapidity being the only thing remarkable. At other times, phthisis is accompanied by very unusual symptoms. It will be found to simulate acute pneumonia, disease of the heart, common fever, with rapid emaciation, &c.

Previously to the researches of Laennec, phthisis was generally deemed fatal. But this illustrious pathologist demonstrated the possibility of cicatrization in cases of tuberculous excavations. Bayle, who was ignorant of this important fact, believed that, in the few cases of apparent cure of phthisis, the disease was merely bronchitis. Auscultation during life, and dissection after death, have proved to a certainty that the said excavations will occasionally heal. Unfortunately this cicatrization is not necessarily followed by the cure of phthisis. For this last to happen, there must be only one excavation, or one mass of tubercles in the lungs, which is rarely the case. There are no facts on record to authorise the conclusion that tubercles in the lungs can be absorbed—therefore the only chance which the patient has (and small is that chance) rests on the evacuation of the tuberculous matter, when once formed, and the healing of the cavern from whence it issued.

VI. TREATMENT OF PHTHISIS. M. Andral lays down two grand indications to pursue—FIRST, to combat the sanguineous congestion (variable in its degree of intensity) which precedes and accompanies the secretion of tuberculous matter—SECONDLY, to remove, if possible, the cause of these tubercular formations. For the fulfilment of the first indication, we have recourse to the antiphlogistic treatment, including revulsives. But it is in that early stage when tubercles are rather apprehended than proved to exist, that the antiphlogistic treatment can be safely made free with. In proportion as the tubercles become multiplied, and especially after they have begun to discharge themselves by the air-passages, we must be cautious of sanguineous emissions. "So far from being beneficial in such cases," says M. Andral, "I have often seen them accelerate the march of the disease." Nothing is more common, in this country, than to freely deplete, locally or generally, for pains in the chest, in cases of tubercular phthisis, by which much mischief is done. M. Andral condemns the use of setons, cauteries, moxas, and other counter-irritants, on the same principle. In the very earliest stage they may be beneficial—but at later periods, he thinks they rather increase the fever by the irritation, and thus tend to develop rather than check the tubercular secretion.

In the *complications* of phthisis, the treatment must be adapted to the nature of the complication. Thus when actual inflammation of the parenchymatous structure of the lungs, or of the bronchial mucous membrane of the pleura obtains, we must deplete, be the consequences what they may, otherwise the disorganization will be rapidly promoted. The strong tendency to inflammation and ulceration of the stomach and bowels, in phthisical patients, should make us cautious in exhibiting tonics and stimulants, in the progress of the disease. M. Andral, indeed, is a violent opposer of tonics in any stage of phthisis; but in this indiscriminate censure we cannot concur. When excavations have occurred, and there is no inflammatory action in the vicinity of these excavations, it is surely reasonable to exhibit nourishing food and tonics, to enable the constitution to cicatrize the cavern. In scrofulous abscesses externally (and the connexion between scrofula and tubercles is strong) we would pursue this course—and we would do the same in cases of pulmonary tuberculation. Besides, the nocturnal perspirations, which so rapidly waste the strength of the patient, are materially checked by the quinine and acids, when judiciously administered. At the same time we are aware of the necessity for a vigilant watch over the symptoms, lest an inflammatory focus be established in some part by the tonics. Neither are we to omit the use of narcotics in moderating the cough. The very succussions of the cough must tend to injure weak lungs, and therefore it is desirable to control it, except when necessary to evacuate the expectoration.

"We see," says M. Andral, "from these facts, how very limited is the therapeutic art in pulmonary phthisis. In a few cases, we can prevent the development of tubercles, or retard their progress. We have no proof that phthisis was ever cured by art. The cicatrization of tuberculous excavations is the work of Nature, in which work she may perhaps be somewhat assisted—or at least not molested in the salutary process. For ages we have been searching after remedies that might combat the disposition to tubercles or remove them when formed—and innumerable specifics have been employed and abandoned in their turn. If I may be permitted to express my opinion, I would say, that this want of success hitherto, forms no good reason for abandoning the pursuit. Among the remedies which have failed, there may some be found, on new and careful trials, to succeed—and we should also experiment with medicinal substances that have not been hitherto employed in this complaint. If the influence of iodine in removing morbid growths of the thyroid gland be demonstrated, which is the case, it is impossible to deny that other medicinal substances may be found capable of modifying other morbid growths or secretions."

In this sentiment, which is equally remote from cheerless scepticism and empirical presumption, we cordially agree with the enlightened author.*

"Air and regimen," says he, "merit great attention in the treatment of phthisis. But what air, what regimen is the best? Here again physicians are far from being of one sentiment. The air of mountains, of forests, and of the sea, &c. has, each in its turn, been praised as beneficial to the consumptive. It appears to me that a *mild temperature* or climate possesses all the advantages which can be reasonably expected from atmospheric influence. Sudden or great aerial vicissitudes—in short, every state or quality of the atmosphere which tends to induce pulmonary congestion, whether acute or chronic, must be injurious to the individual disposed to, or affected with tubercles. To prevent or modify this tubercular disposition, the consumptive have been ordered to breathe air charged with balsamic, resinous, ætherial, sulphureous, and other medicinal substances—as well as with different gases, as oxygen, hydrogen, carbon, &c. Stables, shambles, coal-pits, fens, have been selected as domiciles for the phthisical invalid, but with what success it is not for me to say."

How many volumes have been written on the localities best suited to consumptive patients!! Madeira, Lisbon, Nice, Italy, have annually received their melancholy quota of spectres from the shores of Great Britain—never to return!

Nulla vestigia retro!

While Hastings, Penzance, the Mendip Hills, and some other places have proved the more humble, but perhaps not less salutary domiciles of those who could not afford a more expensive journey or voyage to more brilliant skies.†

---

* There is every reason to believe, from the experiments and statements of Dr. Baron, that iodine itself has a considerable influence in causing the absorption of internal tubercles. We are justified, according to M. Andral's principle, in making more trials with this substance, even before we have recourse to new means.—*Rev.*

† In a little work which now lies before us from the pen of Dr. Myddleton, who appears to have suffered much in his own family from consumption, we find the following passage.

"I have consulted various statistical records, to enable me to ascertain and select the most sanative temporary retreat for consumptive patients, at all seasons of the year, more especially during the cold months; embracing,

"1st. A dry atmosphere of mild temperature, REMOTE FROM THE SEA-SHORE.

"2dly. Complete shelter from the North and North-East winds.

"3dly. Commodious accommodations for visitors.

For our own parts, we are disposed to attach little or no reliance on foreign and southern climates in the cure of tubercles. That a warm and steady atmosphere is useful in preventing or retarding the development of tubercles, there can be little doubt;—but we believe there is no proof that warm climates have cured the disease after the tubercles began to soften down. But the very climates in question, are only serviceable during the Winter and Spring months. Those who go to Italy or Nice must leave them in the Summer and Autumn, and sojourn in Switzerland or some Cisalpine situation. The inconvenience and expence of thus migrating from clime to clime are very great, and probably the chapter of accidents in catching colds, during these migrations, is a full set off against the benefits derived from climate. On all these accounts, we prefer the southern part of England, with English comforts, to the chance of amelioration in warmer regions of the earth.*

---

"4thly. Rational and polished society, so conducive in soothing the mind under corporeal suffering.

"These requisites I have met with at the city of WELLS, on the south side of the Mendip Hills, in the county of Somerset, 20 miles west of Bath. It is not only the most eligible situation (to which my researches have led) in the United Kingdom, but, it would seem, greatly preferable to the South of France, or Italy, Lisbon, or Madeira; for the temperature of those climates is MUCH TOO HIGH, during the summer months, to admit of necessary exercise, independently of being extremely oppressive to pulmonary patients, without any reference to the attendant inconveniences of such removal to invalids, or the painful separation from friends, and the comforts of a native domestic circle; and that too without deriving any sanative advantage whatever from such change of country, which fatal experience has so often lamentably confirmed."

* The Editor of this Journal, during a recent tour through Italy, directed his attention very particularly to the medical topography and climate of those places to which invalids are sent from this country. The more he investigated the subject, the more he was convinced that medical men who send patients, with pulmonary affections, to Italy and the South of France, have a great deal to answer for, if there be retributive justice in another world! Leaving out of the question the passage over the Alps, in Autumn, when atmospheric vicissitudes are abrupt, and the season uncertain, where is the invalid to pitch his tent for the Winter? —Florence is so near the Apennines, that it would be death for a phthisical patient to encounter the tramontane blasts from their snow-clad summits. Even at Naples, you are alternately choaked with the Sirocco from the South, and frozen by the chilling winds from the North. Dr. Johnson went round the *male* wards of the Hospital at Naples (INCURABLES) in company with Mr. Potter, an intelligent English surgeon there, and they counted 84 cases of PHTHISIS in the wards! Allowing that there was an equal number among the females (whom they were not permitted to visit) the sum total would be 168 phthisical cases in a single hospital, not containing more than twelve or fourteen hundred patients! In the great Hospital at Milan, which Dr. J. carefully examined for several days in succession, the proportion of pulmonary and tracheal affections was terrific, and that in the beginning of October, when the Autumn was in its zenith. ROME, from the comparative tranquillity of its atmosphere, the melancholy stillness which reigns over its deserted and pestiferous campagna,

"A weary waste expanding to the skies"—

its distance from the higher ranges of the Apennines, its moderate proximity to

The talented author of the article which we have thus analysed, has overlooked some interesting points, respecting tubercular phthisis, which deserve notice. One of these is, the *communicability* of the disease from one individual to another. Leaving out of the question the popular belief in the contagious character of phthisis entertained in many countries, especially in Italy, we may be permitted to remark that several facts have come within the scope of our observation during the last ten or fifteen years, which convince us that the disorder may be communicated by the breath. We are aware of the difficulty, or even the impossibility of proving this position, beyond the power of cavil—we merely state our own conviction, leaving it for our brethren to conclude according to their own experience. We shall here introduce a passage from Dr. Myddleton's work, in corroboration of what we have advanced.

"That Pulmonary Consumption, derived from hereditary taint can be communicated to persons who are not predisposed to that hitherto ungovernable disease, I have had innumerable unequivocal examples, from attentive observation, with ample opportunities, for more than forty years; but that lamentable occurrence, my experience also teaches me to believe, most confidently, never obtains unless by *direct exposure to the patient's expirations when open ulcers actually exist* in one or both lobes of the lungs, manifested by the expectoration of *pus*. The seeds of the disease are thus sown in the pulmonary organs; tubercles are thence generated in the parenchyma or cellular substance of the lungs by absorption."

Dr. M. goes on to state a considerable number of instances where sleeping in the same bed with, and inhaling the breath of phthisical patients, apparently, and we have no doubt really, induced the disease. But whatever doubt may be thrown on this point, we would advise our professional brethren, for the sake of humanity, to caution those who are well against sleeping in the same bed or room (if it can be avoided) with those who are affected with phthisis, in an advanced stage.

Before concluding this article, we shall just advert to a remedial process strongly advocated by Dr. Myddleton—the direct application of medicinal substances, in the form of impalpable powder, to the lungs themselves—and particularly to the excavations formed by evacuated tubercles. These medicinal substances are, cinchona, myrrh, zinc, and frankincense, introduced by means of an inhaler. The factitious airs applied to the lungs in this way,

---

the Mediterranean, and other circumstances, is, no doubt, much preferable to Florence or Naples for pulmonary invalids. But, from an unbiassed examination of its medical topography, Dr. J. is convinced that, to migrate from the comforts of an English fire-side, and the mild climate of the southern coast of Great Britain, to the cheerless abodes and depressing atmosphere of Rome, is little less than suicidal madness on the part of the patient, and culpable ignorance on the part of the physician who lends his sanction to such a step.

Pisa and Nice appeared to Dr. Johnson as the least exceptionable of the transalpine and cisalpine spots, where phthisical invalids might pass the depth of Winter and the early months of Spring. But the good to be derived from such a residence is a very problematical equivalent to the perils attendant on the journey thither late in Autumn, and the return from thence before the heat of Summer. Dr. J. intends to take a more enlarged view of the subject very soon, and will then have an opportunity of developing his ideas more fully.

by Dr. Beddoes and his followers—and more recently, the application of tar-vapour by Sir Alexander Crichton, have failed of success; and therefore we place no great reliance on the measure employed by Dr. Myddleton. If, indeed, these medicinal agents, which are all stimulants, could be conveyed to the internal surface of an excavation, without touching on any sound part of lung, we think it very probable that a new and salutary action might be occasionally induced in the parietes of the said cavity; but unfortunately not one-twentieth part of any substance introduced by inhalation can be expected to reach the morbid structure—the whole of the remainder being left in contact with the mucous membrane or the air-cells (if a powdery substance reached them) of sound parts. All reasoning would lead us to conclude that such a process would tend to irritate the lungs, and thus keep up an afflux of blood to the seat of disease. That such a process would be detrimental in the long-run, we can have little doubt—and our experience of the effects of tar-vapour corroborates this opinion. Dr. M. insists on the fact, proved by various experiments, that the lungs rapidly absorb substances introduced into them; but we do not see that this alters the case for the better.

We have now brought to a close this exposé of our present state of knowledge respecting pulmonary tubercles. It only wants a plentiful interstitial deposit of *words*, and *cases* to transmute it into a goodly octavo volume, when any young candidate for fame choses to become author.

